# Transoesophageal echocardiography-guided hybrid balloon valvuloplasty for severe pulmonic stenosis in small dogs

**DOI:** 10.4102/jsava.v92i0.2012

**Published:** 2021-01-25

**Authors:** Akiko Uemura, Goya Seijirow, Ryou Tanaka, Meriç Kocaturk, Zeki Yilmaz

**Affiliations:** 1 Laboratory of Veterinary Surgery, Department of Clinical Veterinary Medicine, Division of Veterinary Research, Obihiro University of Agriculture and Veterinary Medicine, Hokkaido, Japan; 2 Department of Veterinary Surgery, Faculty of Veterinary Medicine, Tokyo University of Agriculture and Technology, Tokyo, Japan; 3 Department of Internal Medicine, Faculty of Veterinary Medicine, Uludag University Bursa, Görükle, Turkey

**Keywords:** dog, balloon valvuloplasty, small breed, thoracotomy, transoesophageal echocardiography

## Abstract

Pulmonic stenosis (PS) is the most common congenital heart disease in dogs and is commonly seen in small breeds, such as Chihuahuas. Conventional treatments have limitations specific to small dogs, including the invasive nature of open-heart surgery and size limitations in percutaneous balloon valvuloplasty. Here, transoesophageal echocardiography (TEE)-guided balloon valvuloplasty via thoracotomy was performed for three small dogs with PS. The procedure was feasible in all cases, including those for which percutaneous treatment was not an option. Although the procedure is invasive, because of the need for thoracotomy, it is one of the treatment options that may be effective for PS, especially in small dogs, as it allows visualisation of the pulmonary artery lesion without relying on the experience of the surgeon.

## Introduction

Pulmonic stenosis (PS) is the most common congenital heart disease in dogs (Schrope 2015). Surgical options for PS in dogs include dilation of the right ventricular outflow tract via cardiopulmonary bypass (Soda et al. 2009) and percutaneous balloon valvuloplasty (Bussadori et al. 2001). The most common type of PS is valvular PS. Valvular PS can be differentiated into two main types (Bussadori et al. 2000), type A and type B. With type A PS, the annular size is normal. Various degrees of leaflet thickening can occur, from incomplete separation of the commissures to almost complete fusion. The condition causes a systolic doming of the valve (A ‘windsock’-type image), frequently involving eccentric valvular opening with varying degrees of reduced cross-sectional area. Poststenotic dilatation of the pulmonary trunk is invariably present, with varying degrees of severity. Type A PS is ideally suited for balloon valvuloplasty procedures. With type B PS, the pulmonary ostium becomes hypoplastic, with varying degrees of valvular leaflet thickening and immobility but little commissural fusion. The main pulmonary trunk is also often hypoplastic, and rarely has poststenotic dilatation. Soda et al. surgically dilated the diameter of a pulmonary artery stenosis by patching the right ventricular outflow tract. Although dilation of the right ventricular outflow tract is effective in resolving stenosis, the use of an artificial heart–lung system is associated with the risk of multi-organ failure (Mei et al. 2018). This procedure is particularly complicated, with increased surgical risks, in small, light-weight dogs. Percutaneous balloon valvuloplasty is less invasive and can be effective in the treatment of valvular stenosis. However, as the insertion of the device is dependent on the diameter of the vessels, it can be difficult to change the direction of the device in narrow, hypertrophied ventricles. There have been multiple reports in human medicine about the size of balloons that can be used effectively and safely in balloon valvuloplasty for PS. In general, it is significantly more effective and safe if the balloon : annulus diameter ratio (BAR) is in the range of 1.2 to 1.4 (Janus et al. 2006; Radtke et al. 1986). The sizes of balloons used in the cases described in this report were selected to include balloons with BARs in the range of 1.2 to 1.4.

In the present study, balloon valvuloplasty via thoracotomy was performed for severe valvular PS in three small dogs for which percutaneous balloon valvuloplasty was deemed too difficult to perform.

## Case presentation

### Case 1

**FIGURE 1:**
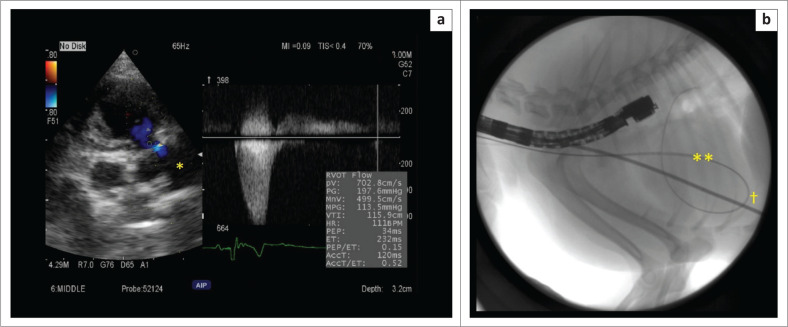
Case 1; (a) Right parasternal short-axis view at the level of the aorta and pulmonary artery: A transthoracic echocardiogram showed pulmonary stenosis (pulmonic velocity 7.0 metres per second, peak systolic gradient 197.6 mmHg) on day 1, pulmonary artery (*); (b) Surgical fluoroscopy image on day 6 (first surgery). The guidewire was advanced to the right ventricular apex and retroflexed to reach the site of stenosis in the main pulmonary artery. However, the balloon could not follow the guide wire (**) because it was unable to turn at the sharp curve at the right apex (†).

A 1-year-old male Pomeranian, weighing 4.1 kilograms (kg), presented with occasional fainting whilst walking. The dog was suspected to have a congenital heart disease after a heart murmur was heard by a local veterinarian. The dog was referred to the Tokyo University of Agriculture and Technology (TUAT) Animal Medical Center for further investigation and treatment. Thoracic auscultation revealed a grade 5/6 left basilar systolic murmur. Heart rate (HR) and blood pressure (BP) measured on day 1 of admission were 120 beats per minute (bpm) and 160/121 (135) (systolic/diastolic [mean]) mmHg (using an oscillometric method), respectively. A transthoracic echocardiogram (TTE) showed severe valvular PS (pulmonic velocity [PV] 7.0 metres per second (m/s), peak systolic gradient [PSG] 197.6 millimetre of mercury [mmHg]) (Figure 1a), with persistent left-sided cranial vena cava. The classification of PS was type B. The annulus of the pulmonary artery was 8.6 mm. See Table 1 for additional echocardiographic information for all three dogs.

**TABLE 1:** Echocardiographic findings for the three dogs.

Patient	PS type	Pre/post OP	PS velocity	PSG	Classification†	TVR	RVH‡	Other heart disease
Case 1	B	Pre	7.0 m/s	197.6 mmHg	Severe	Absent	Moderate	Absent
		Post	6.2 m/s	153.8 mmHg	Severe	Absent	Moderate	Absent
Case 2	B	Pre	7.1 m/s	202.0 mmHg	Severe	Absent	Moderate	Absent
		Post	6.0 m/s	144.0 mmHg	Severe	Absent	Moderate	Absent
Case 3	A	Pre	6.0 m/s	144.0 mmHg	Severe	Absent	Mild	ASD-like condition§
		Post	2.7 m/s	28.5 mmHg	Mild	Absent	Mild	Absent

PS, pulmonary stenosis; PSG, peak systolic gradient; TVR, tricuspid valve regurgitation; ASD, atrial septal defect; OP, operation; RVH, right ventricular hypertrophy.

† , Mild stenosis: peak gradient from 20 mmHg to 49 mmHg (2.25 m/s – 3.5 m/s); moderate stenosis: peak gradient from 50 mmHg to 80 mmHg (3.5 m/s – 4.5 m/s); severe stenosis: peak gradient more than 80 mmHg (more than 4.5 m/s) (Bussadori et al. 2000).

‡ , The degree of RVH was recorded as mild, moderate or severe. Mild hypertrophy was defined as subjectively increased right ventricular wall thickness with absence of paradoxical motion of the interventricular septum on the right parasternal short-axis view. Moderate hypertrophy was defined as mild paradoxical motion of the interventricular septum. Severe RVH was defined as marked paradoxical motion of the septum on this view (Johnson et al. 2004).

§ , ASD-like condition: this case was associated with an ASD-like condition as a result of the increased load on the right heart caused by PS, resulting in an open foramen ovale state.

Percutaneous valvuloplasty was initially attempted via the right jugular vein on day 6. However, dilation was not performed because the balloon (size: 10 mm diameter × 30 mm length) (Balloon dilation catheters, Infiniti Medical LLC, United States) could not be turned along the stiff guide wire at the right ventricular apex (Figure 1b). The dog was discharged the following day.

**FIGURE 2:**
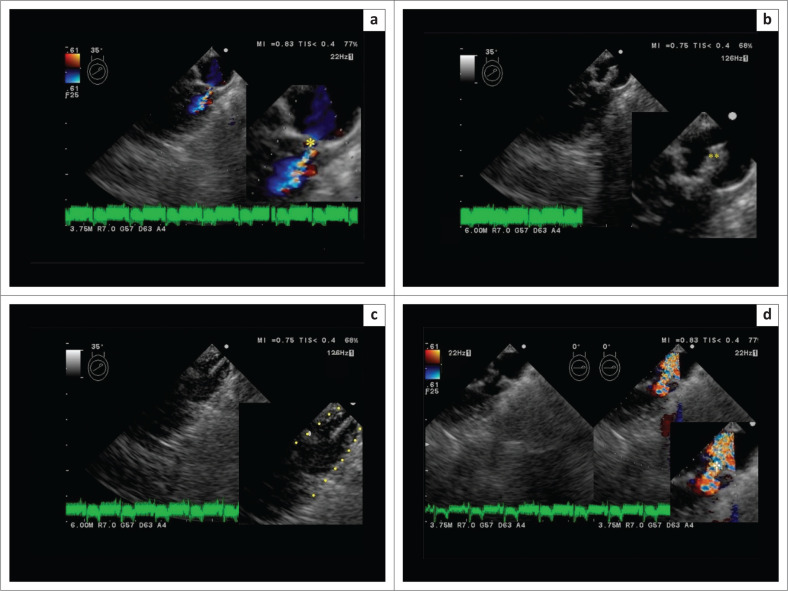
A transoesophageal echocardiography image showing the surgical procedure (an enlarged view is shown at the lower left). Case 1, TEE-guided hybrid balloon valvuloplasty was performed via thoracotomy on day 27; (a) Before hybrid balloon valvuloplasty, colour flow Doppler (*: the stenotic portion of the pulmonary stenosis); (b) A 5-Fr sheath was inserted in the pulmonary stenosis site (**), B-mode; (c) The balloon was inflated at the pulmonary stenosis site (dotted line: outline of the inflating balloon catheter), B-mode; (d) Following hybrid balloon valvuloplasty, the stenotic portion of the pulmonary stenosis was dilated (†). (Left, B-mode; Right, colour flow Doppler).

The dog was returned to the hospital on day 27 and admitted again. Transoesophageal echocardiography (TEE)-guided hybrid balloon valvuloplasty was performed via thoracotomy on the same day (Figure 2a). The fourth intercostal space on the dog’s left side was opened. A purse-string suture was placed on the right side of the cardiac apex. Instead of making an incision in the heart, the heart was punctured with an external and an internal mantle to create a puncture hole using the Seldinger technique, which allows the needle tip to be judged to be in the lumen of the heart by blood flowing backward from the mantle. A 5 Fr sheath (length: 3.centimetres [cm]) (Ultra high-flow sheath, Medikit Co. Ltd.) was inserted into the punctured hole, from the right apex of the heart into the pulmonary artery (Figure 2b) and monitored using TEE (UST-5293S-5; Hitachi-Aloka Medical, Ltd., Japan) to check its location. A 0.035 guide wire was inserted, delineated and positioned as it was passed through the stenotic lesion in the pulmonary artery. Then, the balloon (10 mm diameter × 30 mm length [TYSHAKU II]) was positioned under TEE guidance. The balloon continued to be moved under TEE guidance, following the guidewire through the stenosis. The balloon was inflated five times whilst maintaining visualisation of the stenosis via TEE (Figure 2c, d). The BAR was 1.2. After ensuring that the dog did not develop arrhythmia, the chest wall was closed using the conventional technique. The operation time, from incision to chest closure, was 54 min. The dog recovered from anaesthesia without any problems. The dog was discharged on day 30. Transthoracic echocardiogram performed on day 78 showed a reduction in PS, with a PV decrease to 6.2 m/s (PSG 153.8 mmHg). On day 188, the dog exhibited no clinical symptoms, such as fainting. At 973 days after the second surgery, his general condition, including his vigour and appetite, was good.

### Case 2

A 4-month-old female Chihuahua, weighing 1.7 kg, presented with occasional exercise intolerance. The dog was suspected to have a congenital heart disease after a heart murmur was heard by a local veterinarian, who suggested the dog be referred to the TUAT Animal Medical Centre for further investigation. Thoracic auscultation revealed a grade 5/6 left basilar systolic murmur. On day 1 of admission, HR and BP were 233 bpm and 155/109 (129) mmHg, respectively. Chest radiographs showed right ventricular enlargement and post-stenotic dilatation findings in the pulmonary artery. Transthoracic echocardiogram showed severe valvular PS (PV 7.1 m/s, PSG 202.0 mmHg). The classification of PS was type B. The annulus of the pulmonary artery was 8.3 mm. Transoesophageal echocardiography-guided hybrid balloon valvuloplasty was performed as with Case 1, on day 10. A balloon (10 mm diameter × 30 mm length [TYSHAKU II]) was inserted and inflated three times. The BAR was 1.2. No sheath was used. She recovered from anaesthesia uneventfully. The operation time was 88 min. She was discharged the day following surgery. On day 148, TTE showed a reduction in PS, with a PV decrease to 6.0 m/s (PSG 144.0 mmHg). On day 750, there was no change in PV (6.0 m/s) (PSG 144.0 mmHg), and she exhibited no clinical symptoms. At 894 days post-operatively, Case 2 exhibited no clinical symptoms and a good general condition, such as her vigour and appetite.

### Case 3

An 8-month-old male Bichon Frise, weighing 2.2 kg, presented with dyspnea and cyanosis. The local veterinarian suspected a severe congenital heart disease and had prescribed 2.0 milligrams (mg)/kg/day of furosemide and 0.25 mg/kg b.i.d. of pimobendane. However, the dog presented with dyspnea and cyanosis on room air and needed to be kept in a room maintained at 40% O_2_. He was referred to the TUAT Animal Medical Center for further investigation and treatment. Thoracic auscultation revealed a grade 4/6 left basilar systolic murmur.

On day 1,TTE showed severe valvular PS (PV 6.0 m/s, PSG 144.0 mmHg). The classification of PS was type A. The annulus of the pulmonary artery was 6.9 mm. The cardiac condition was complicated by a right cardiac overload caused by PS, which resulted in an open foramen ovale and consequently an atrial septal defect (ASD)-like condition. Transoesophageal echocardiography-guided hybrid balloon valvuloplasty was performed as in Case 1 and 2, on day 15. A 5-Fr sheath was inserted. A balloon (8 mm diameter × 20 mm length) was inserted and inflated four times. The BAR with this first balloon was 1.2. Then, the balloon was replaced with different sized balloons. The second balloon (10 mm diameter × 30 mm length) was inflated three times, and the third balloon (12 mm diameter × 30 mm length) was inflated four times. He recovered from anaesthesia uneventfully. The operation time was 91 min. On day 16, TTE showed a reduction in PS, with a PV decrease to 2.7 m/s (PSG 28.5 mmHg), and the ASD disappeared as the capacity overload in the right side of the heart was reduced. On the same day, he was discharged. On day 101, although he had been on oral medication, he was active without any clinical symptoms on room air. Case 3 died as a result of an accident at home unrelated to his cardiac disease, but he had a good appetite and a very active life at home until the day of his death (140 days postoperative).

### Ethical consideration

No approval was required as this case report describes clinical cases seen at the Faculty of Veterinary Medicine, Tokyo University of Agriculture and Technology.

## Discussion

Surgical treatment is recommended for dogs with severe PS that present with clinical symptoms and have a pressure gradient of at least 75 mmHg (Johnson et al. 2004). Surgical dilation of the right ventricular outflow tract is effective for resolving PS (Fujiwara et al. 2012). Although some studies have reported the surgical treatment of PS via cardiopulmonary bypass in small dogs (Fujiwara et al. 2012), the procedure requires a high level of technical skill and is particularly risky for small dogs. Thus, the procedure is only feasible at certain institutions.

Using the Brock method, the right ventricular outflow tract is dilated by inserting a device such as forceps directly into the heart following thoracotomy. This procedure facilitates the placement of the device into the site of stenosis (Brock 1948) and has been applied in small hearts, such as those of newborns and infants (Vogel et al. 1990). However, there are safety concerns associated with the use of a steel instrument to blindly dilate the heart chambers, and the surgical outcome is often inconsistent because it depends on the experience of the surgeon.

Percutaneous balloon valvuloplasty is less invasive and is effective for the treatment of valvular stenosis (Belanger et al. 2018; Goya et al. 2018; Ramos, Monteiro-Steagall & Steagall 2014). However, PS is common in several small breeds (Pelosi & Orton 2017). As demonstrated in the present case, it can sometimes be challenging to change the direction of a device in narrow heart chambers. Pulmonic balloon valvuloplasty using the femoral vein approach does not require a 180° rotation at the right ventricular apex. However, in small breed puppies, the femoral veins are narrow, and it is sometimes difficult to insert a sheath. There is also the physical distance from the puncture site to the pulmonary artery. The catheter track from the caudal vena cava through the right ventricle to the pulmonary artery is not simple either. The procedure has several other limitations, including an increase in the exposure to fluoroscopic irradiation for surgeons and the patient, as well as the complexity of and need for advanced wiring techniques during the remote insertion of the device into the site of stenosis.

As demonstrated in the present study, TEE-guided balloon valvuloplasty via thoracotomy is advantageous because it overcomes the limitations discussed here. Firstly, the procedure involves direct insertion of the sheath, guidewire and balloon catheter into the right ventricular outflow tract via the right ventricular apex, to give easy access to the stenotic valve. This facilitates the movement of the guidewires and catheters within the heart chambers, as well as allowing multiple balloon inflations and exchange of the catheters. The procedure also facilitates interventional radiology procedures and does not require a high level of technical skill. Furthermore, there is no risk of radiation exposure for the surgeon or the patient. Transoesophageal echocardiography guidance allows for real-time visualisation of the site of stenosis and monitoring of dilation during the procedure, allowing for accurate dilation of the site without relying on the experience of the surgeon. This presents an educational opportunity because each individual in the operating room can share the same information at the same time. The procedure is highly reproducible compared with the reproducibility of surgical techniques that depend on the experience of the surgeon. Following this surgery, in Case 3 of PS type A, severe PS became mild PS because of a significant decrease in pulmonary artery flow velocity and PSG. The therapeutic effect of the treatment was significant in terms of TEE, and the dog’s clinical symptoms disappeared. On the other hand, Case 1 and Case 2, which were preoperatively diagnosed as type B PS, remained severe PS, but both showed resolution of their clinical symptoms. In type B PS, the effect of treatment using balloon valvuloplasty is more limited than of the effect seen for type A (Bussadori et al. 2001). A similar tendency was observed here when using this method, but it has been suggested that it results in mild resolution of clinical signs.

The limitations of this procedure include the following. Firstly, although it is not as invasive as open-heart surgery, it is more invasive than a percutaneous approach because it requires thoracotomy. Secondly, the distance between the site of sheath insertion and stenosis is relatively short. As a result, the sheath may be extracted by mistake during the procedure, which further increases the risk of bleeding. A potential complication of pulmonic balloon valvuloplasty is the rupture of an aberrant right coronary artery, which is a concern in patients with this malformation. The risk of this complication is unlikely to be reduced with this procedure. Thus, as with conventional pulmonic balloon valvuloplasty, extreme caution must be exercised in the presence of abnormal right coronary arteries in susceptible breeds, especially breeds with a high incidence of disease.

In the present study, TEE-guided balloon valvuloplasty via thoracotomy was performed for severe PS in small dogs. It resulted in rapid recovery from anaesthesia with no postoperative complications, and it improved the symptoms associated with PS.

Collectively, the present findings suggest that TEE-guided balloon valvuloplasty via thoracotomy is one of the treatment options that is effective in alleviating symptoms associated with PS, particularly in small dogs with severe PS.
